# 1-methylnicotinamide and its structural analog 1,4-dimethylpyridine for the prevention of cancer metastasis

**DOI:** 10.1186/s13046-016-0389-9

**Published:** 2016-07-13

**Authors:** Agnieszka Blazejczyk, Marta Switalska, Stefan Chlopicki, Andrzej Marcinek, Jerzy Gebicki, Marcin Nowak, Anna Nasulewicz-Goldeman, Joanna Wietrzyk

**Affiliations:** Hirszfeld Institute of Immunology and Experimental Therapy, Polish Academy of Sciences, Weigla 12, 53-114 Wroclaw, Poland; Chair of Pharmacology, Jagiellonian University, Medical College, Grzegórzecka 16, 31-531 Krakow, Poland; Jagiellonian Center for Experimental Therapeutics (JCET), Jagiellonian University, Bobrzynskiego 14, 30-348 Krakow, Poland; Lodz University of Technology, Zeromskiego 116, 90-924 Lodz, Poland; Wroclaw University of Environmental and Life Sciences, Norwida 31, 50-375 Wroclaw, Poland

**Keywords:** 1-methylnicotinaimide chloride, 1,4-dimethylpyridinium chloride, Metastasis, Angiogenesis, Combined therapy, Breast cancer

## Abstract

**Background:**

1-methylnicotinamide (1-MNA), an endogenous metabolite of nicotinamide, has recently gained interest due to its anti-inflammatory and anti-thrombotic activities linked to the COX-2/PGI_2_ pathway. Given the previously reported anti-metastatic activity of prostacyclin (PGI_2_), we aimed to assess the effects of 1-MNA and its structurally related analog, 1,4-dimethylpyridine (1,4-DMP), in the prevention of cancer metastasis.

**Methods:**

All the studies on the anti-tumor and anti-metastatic activity of 1-MNA and 1,4-DMP were conducted using the model of murine mammary gland cancer (4T1) transplanted either orthotopically or intravenously into female BALB/c mouse. Additionally, the effect of the investigated molecules on cancer cell-induced angiogenesis was estimated using the matrigel plug assay utilizing 4T1 cells as a source of pro-angiogenic factors.

**Results:**

Neither 1-MNA nor 1,4-DMP, when given in a monotherapy of metastatic cancer, influenced the growth of 4T1 primary tumors transplanted orthotopically; however, both compounds tended to inhibit 4T1 metastases formation in lungs of mice that were orthotopically or intravenously inoculated with 4T1 or 4T1-luc2-tdTomato cells, respectively. Additionally, while 1-MNA enhanced tumor vasculature formation and markedly increased PGI_2_ generation, 1,4-DMP did not have such an effect. The anti-metastatic activity of 1-MNA and 1,4-DMP was further confirmed when both agents were applied with a cytostatic drug in a combined treatment of 4T1 murine mammary gland cancer what resulted in up to 80 % diminution of lung metastases formation.

**Conclusions:**

The results of the studies presented below indicate that 1-MNA and its structural analog 1,4-DMP prevent metastasis and might be beneficially implemented into the treatment of metastatic breast cancer to ensure a comprehensive strategy of metastasis control.

**Electronic supplementary material:**

The online version of this article (doi:10.1186/s13046-016-0389-9) contains supplementary material, which is available to authorized users.

## Background

1-methylnicotinamide (1-MNA), an endogenous metabolite of nicotinamide (NA), is synthetized in the reaction of nicotinamide N-methyltransferase (NNMT), an enzyme expressed predominantly in the liver, where it plays a pivotal role in the N-methylation of NA and possibly other pyridine compounds [1]. However, NNMT expression has also been reported in a number of other types of cells and organs, including rapidly proliferating cancer cells [2–4] or the brains of the patients suffering from Parkinson’s disease [5]. For decades it has been nicotinamide, a substrate for the NNMT reaction that has attracted attention as a possible therapeutic agent endowed with pharmacological activities [6–9]. Meanwhile, 1-MNA has been considered an inactive metabolite [10]. Only recently has some evidence for the possible therapeutic application of 1-MNA emerged. The first signals for its pharmacological potential date back to 2003, when Gebicki et al. described its anti-inflammatory activity in skin disorders such as acne or contact dermatitis [11], which was further confirmed in an additional study involving patients with rosacea [12]. In turn, studies by Chlopicki et al. demonstrated that the mechanisms of action of 1-MNA involve the activation of PGI_2_ release driven by cyclooxygenase 2 (COX-2) [13]. PGI_2_ releasing capacity of 1-MNA was later shown to afford not only anti-thrombotic [13] but also fibrinolytic [14], anti-inflammatory [11, 15] and gastroprotective [16] effects. Interestingly, 1-MNA did not directly either affect the activity of leucocytes [15, 17] or release PGI_2_ in the perfused rat hindquarters model [18]. Still, 1-MNA, due to its PGI_2_ releasing capacity, might serve as a hepatoprotective agent that protects against concanavalin A induced liver injury [19] through the downregulation of interleukin-4 (IL-4) and tumor necrosis factor-α signalization (TNF-α) [20]. In addition to its anti-platelet, anti-thrombotic and anti-inflammatory activities, 1-MNA has also been shown to restore endothelial function in diabetic hypertriglycemic rats [21], as well as to improve endothelial function in humans [22].

Given the reports demonstrating that PGI_2_ displays anti-metastatic activity [23, 24], and the PGI_2_ releasing activity of 1-MNA [13], the potential application of exogenous 1-MNA to prevent metastatic cancer seems to be justified. However, currently only limited reports on the in vivo anti-cancer activity of 1-MNA are available, including the daily use of 1-MNA in mice bearing murine leukemia L1210 that resulted in about a 50 % lifespan increase [25] or the disclosure of the use of 1-MNA in the prevention of experimental metastases formation [26].

In his patent application Gebicki et al. [26] discloses also the use of the 1-MNA’s analog, 1,4-dimethylpyridine (1,4-DMP), another pyridine compound naturally occurring in roasted coffee seeds. 1,4-DMP is believed to be at least partially responsible for health-beneficial effects of coffee and as such has been shown to inhibit thrombus formation in vivo. As it has been observed for 1-MNA, the antihrombotic activity of 1,4-DMP depends on the prostacyclin-related mechanisms [27]. What is more, results of our preliminary studies have shown that 1,4-DMP inhibits metastases formation in the model of spontaneously metastasizing murine mammary gland cancer (4T1) [28]. Nevertheless, none of the mentioned reports provide any detailed evidence of the impact revealed by 1-MNA or 1,4-DMP on the growth and metastasis of solid tumors.

Metastasis is a complex process comprising several discrete steps that result in the formation of secondary lesions localized at a distance from the primary tumor. Due to their characteristics, metastases often fail to respond to standard chemotherapy and appear to be inaccessible for irradiation or surgical treatment. Therefore, their development usually leads to a dramatic deterioration in survival prognosis, which, in turn, justifies the common urge for modern therapeutic regimes enabling the control of metastatic dissemination. Since it is undeniable that successful metastasis relies on the complex interactions between cancer cells and host body tissues, one can expect that selective interference with such an interaction would reduce metastatic potential. The phenomena occurring in a host body tissues that have been suggested to beneficially influence cancer progression comprise, for example, tumor vessel formation, tumor immune editing and also tumor cell induced platelet aggregation (TCIPA). The latter seems to be of particular interest, as it not only promotes cancer metastasis but also increases patients mortality due to cardiovascular incidents [29]. Nevertheless, platelets undoubtedly contribute to metastasis efficiency as they physically shield migrating cancer cells in the bloodstream, facilitate immune evasion [30, 31], and promote cancer cell adhesion to the endothelium of the microvasculature in a target tissue [32, 33]. Thus, platelets appear to be a promising alternative target for metastasis prevention. Inhibition of metastases development by anti-platelet pharmacotherapy has been previously studied, and to date multiple data on the anti-metastatic potential of anti-platelet agents have been reported [34–37]. PGI_2_is the most potent endogenous anti-platelet agent that, together with its analogs, has been shown to prevent cancer metastasis [23, 38–42]; however, the unfavorable short lifetime of the parent molecule and the hypotensive action of its potent analogs limit their application.

Given the PGI_2_-related mechanism of action of 1-MNA and 1,4-DMP, we decided to investigate whether these compounds may possess any significant anti-cancer activity in the model of highly metastatic murine mammary gland cancer (4T1). The secondary aim of the presented study was to determine the possibility to utilize both of the compounds in a therapeutic regimen additionally comprising cyclophosphamide.

## Methods

### Compounds

1-methylnicotinamide (1-MNA) and 1,4-dimethylpyridinium (1,4-DMP) were used in the form of chlorides provided by the Lodz University of Technology. Prior to use, pyridinium salts were diluted in drinking water such that mice received 100 mg/kg/day of the drug. Cyclophosphamide (Endoxan) was purchased from Baxter Oncology GmbH, (Germany). All the drugs were administrated according to the dosing and administration schedules provided in Table 1 and corresponding graphs below.

**Table 1:** Drugs, doses and therapeutic regimens applied in the presented studies

Experimental model	Drug	Route of administration	Dose	Treatment regimen
Experimental metastasis (Fig. 1)	1-MNA	*Per os* in drinking water	100 mg/kg/day	Treatment initiated 7 days prior cancer transplantation and continued to the end of the experiment
	1,4-DMP			
Spontaneous metastasis (single drug treatment) (Fig. 2)	1-MNA	*Per os *in drinking water	100 mg/kg/day	Continuously from the 7th day of the experiment to the end of the experiment
	1,4-DMP			
Matrigel plugs (Fig. 3)	1-MNA	*Per os* in drinking water	100 mg/kg/day	Continuously starting from the day of matrigel plugs transplantation
	1,4-DMP			
	Cyclophosphamide	intraperitoneally	25 mg/kg/day	On days 0, 3, 5
Spontaneous metastasis (combined treatment) (Fig. 4 and 5)	1-MNA	*Per os* in drinking water	100 mg/kg/day	Continuously from the 1st day of the experiment to the end of the experiment
	1,4-DMP			
	Cyclophosphamide	Intraperitoneally	25 mg/kg/day	From the 7th day of the experiment, 3 times each week

### Mice

7/8-week-old BALB/c female mice were purchased from the Center of Experimental Medicine, Medical University of Bialystok, Poland. 7/8-week-old BALB/c Nude female mice were provided by Charles Rivers Laboratories (Germany). All experiments were performed according to the *Interdisciplinary Principles and Guidelines for the Use of Animals in Research, Marketing and Education* issued by the New York Academy of Sciences’ Ad Hoc Committee on Animal Research and were approved by the 1st Local Committee for Experiments with the Use of Laboratory Animals, Wroclaw, Poland (Table 2).

**Table 2:** Strains and number of mice used in the experiments

Experimental model	Mouse strain	No. of mice/group	Total No. of used mice
Experimental metastasis (Fig. 1)	BALB/c Nude	7	21
Spontaneous metastasis (single drug treatment) (Fig. 2)	BALB/c	10	30
Matrigel plugs (Fig. 3)		9	36
Spontaneous metastasis (combined treatment) (Figs. 4 and 5)		10	50

### Cell culture and transplantation

The mouse mammary adenocarcinoma 4T1 cells were obtained from the American Type Culture Collection (ATCC, USA). Cells were cultured in RPMI 1640 (IIET, Poland) with Opti-MEM® (Life Technologies, USA) (1:1 v/v) medium with 5 % fetal bovine serum (HyClone, Thermo Fisher Scientific Inc. UK), supplemented with 4.5 g/L glucose, 2 mM glutamine, 1.0 mM sodium pyruvate (all from Sigma-Aldrich, Germany) and antibiotics (penicillin and streptomycin – Polfa Tarchomin, Poland). The mouse mammary adenocarcinoma 4T1-luc2-tdTomato cell line stably expressing the firefly luciferase gene and tdTomato fluorescent protein was obtained from Caliper Life Sciences Inc. (USA). Cells were cultured in RPMI 1640 + Gluta-MAX™ medium (Life Technologies, USA) supplemented with 10 % fetal bovine serum (Sigma-Aldrich, Germany) and antibiotics (penicillin and streptomycin – Polfa Tarchomin, Poland). Both cell line cultures were maintained at 37 °C in a humidified atmosphere with 5 % CO_2_.

Irrespective of the tumor model applied prior to the transplantations, all cells were trypsinized (IIET, Poland), centrifuged (200 g, 4 °C, 5 min) and counted. For the model of spontaneous metastasis, 4T1 cells were resuspended in Hank’s Balanced Salt Solution (HBSS; IIET, Poland) such that a suspension of 3 × 10^5^ 4T1 cells in 0.05 ml of HBSS was inoculated into the mammary gland of female BALB/c mice. For the model of experimental metastasis, 4T1-luc2-tdTomato cells were resuspended in HBSS such that a suspension of 3 × 10^5^ 4T1-luc2-tdTomato cells in 0.1 ml HBSS was injected into the tail vein of each female BALB/c Nude mice. For the model of matrigel plugs, 4T1 cells were resuspended in the mixture of matrigel (BD Matrigel Basement Matrix High Concentration, Becton, Dickinson and Company, USA) and HBSS (5:1) such that 1 × 10^4^ cells suspended in 0.3 ml mixture was injected subcutaneously on the right flank of each female BALB/c mice.

### Estimation of the anti-tumor activity

When tumors became palpable, their maximum length and width were measured 3 times a week, and the tumor volume was calculated according to the following formula:

$$ TV = \frac{1}{2}\times {a}^2\times b $$

Where TV is the tumor volume, a is the shorter diameter and b is the longer diameter. To compare tumor growth inhibiting activity between the experimental groups, tumor growth inhibition values were estimated using the following formula:

$$ TGI=100-\frac{T{V}_N}{T{V}_C}\times 100 $$

Where TGI is the tumor growth inhibition value, TV_N_ is the mean tumor volume calculated for the studied experimental group and TV_C_ is the mean tumor volume calculated for the control group of animals.

### Evaluation of the anti-metastatic effect

To determine the anti-metastatic activity of the compounds, the lungs of tumor bearing mice were excised, weighed and transferred into 5 % solution of buffered formalin. After tissue fixation, metastatic foci were counted visually. In the model of experimental metastasis, in vivo visualizations of the metastatic foci localized in lungs were performed every third day, starting from the tenth day of the experiment using an In vivo MS FX PRO system (Carestream Health INC., USA). In brief, about ten minutes before imaging, D-luciferine potassium salt (Synchem INC., Germany) was administered to each mouse intraperitoneally at the dose of 150 mg/kg. Then, animals were anesthetized with a 3–5 % (v/v) mixture of isoflurane (Forane, Abbott Laboratories, USA) in synthetic air (200 ml/min). Anesthesia was maintained by means of individual masks providing a 1.5–2 % (v/v) mixture of isoflurane and synthetic air. Visualization was carried out using the following settings: for X-Ray t = 2 min, f-stop = 5.57, FOV = 198.6; for luminescence capture t = 3 min, binning 2 × 2, f-stop = 5.57, FOV = 198.6. Images were analyzed with Carestream MI SE software (Carestream Health INC., USA). The intensity of the luminescent signal is presented as the sum intensity of the region of interest and expressed in arbitrary units [a.u.].

### Evaluation of tumor angiogenesis

The influence of 1-MNA and 1,4-DMP on tumor angiogenesis was evaluated in the model of spontaneously metastasizing 4T1 tumors and confirmed in the model of matrigel plugs. Analyzes were performed by means of ultrasound imaging of tumor perfusion or immunohistochemistry staining of platelet endothelial cell adhesion molecules (PECAM-1; CD31) in the matrigel matrix, respectively. For the ultrasound imaging, the MicroMarker™ Contrast Agent (VisualSonics, Canada) was prepared according to the manufacturer’s instructions. Animals were anesthetized with the 3–5 % (v/v) mixture of isoflurane (Forane, Abbott Laboratories, USA) in synthetic air (200 ml/min) and placed on an animal handling station equipped with an individual mask providing a 1.5–2 % (v/v) mixture of isoflurane and synthetic air. The position of the handling station was adjusted so that the central section of the tumor was being visualized and the contrast agent was injected into the tail vein of the animal. The signal of the contrast marker accumulating in the tumor mass was recorded using a probe of 13–24 Mhz frequency (MS250, VisualSonics, Canada). Next, the wash-in rate was calculated using Vevo LAB 1.7.1. Software (VisualSonics, Ontario, Canada).

Immunohistochemistry was performed in matrigel plugs (isolated from mice on the 7th day after the transplantation) which were fixed in buffered formalin and then cut into 4-μm-thick sections that were subsequently dewaxed with xylene and rehydrated in a gradient of ethanol. For antigen retrieval, sections were heated in a water bath at 96 °C for 20 min with EnVision™ FLEX Target Retrieval Solution, High pH (Dako®, Carpinteria, USA). Endogenous peroxidase activity was quenched in EnVision™ FLEX Peroxidase-Blocking Reagent for 5 min. Thereafter, sections were incubated with primary antibody against PECAM-1 (diluted in 1:50 ratio). Subsequently, sections were washed in EnVision™ FLEX Wash Buffer and detection system EnVision™ FLEX/HR SM802 was added for 30 min at room temperature. The reaction was developed using 3,3-diaminebezidine tetrahydrochloride solution (DAB, EnVision™ FLEX DAB+ Chromogen (DAKO®, Carpinteria, USA). Finally, sections were washed in distilled water and nuclei were counterstained in hematoxylin, preparations were dehydrated in an alcohol gradient and coverslip mounted. Mean vessel density was evaluated by counting PECAM-1-positive vessels in 5 different fields of view at 200× magnitude.

### Platelet activation status

Blood samples from the experimental animals were collected on the last day of the experiments. Samples were collected into tubes containing 0.05 ml of 5 % ethylenediaminetetraacetic acid (EDTA) solution (Sigma-Aldrich, Germany). Then, blood plasma was obtained by centrifugation (2000 g, 15 min, 4 °C) and stored at −80 °C until further analyzed. Prostacyclin generation in the treated mice was determined by the quantification of plasma 6-keto-prostaglandin F1α (6-keto-PGF1α) levels. Platelet activation status was estimated on the basis of thromboxane B_2_ (TXB_2_), von Willebrand factor (vWF) and soluble P-selectin plasma concentrations. All analyzes were conducted using commercial ELISA kits available from Cusabio Biotech CO., Ltd (Wuhan, China), according to the manufacturer’s instructions.

### E-cadherin/N-cadherin expression in tumor tissue

Protein expression in 4T1 tumor tissue was analyzed according to the standard Western blot procedure [43]. In brief, samples of tumor tissue collected and immediately frozen on the last day of the experiments were homogenized in RIPA Buffer (Sigma-Aldrich, Germany) using a FastPrep®-24 MP Bio device (Mp Biomedicals LLC., USA) with the following settings: CP 24x2, 6 m/s, 40 s. Protein content in all samples was analyzed using a Bio-Rad Protein Assay (Bio-Rad Laboratories Inc., USA) according to the manufacturer’s protocol. Samples containing 100 μg of protein were separated on the pre-cast 4–20 % gradient gels (Bio-Rad Laboratories, Inc., USA) and transferred onto 0.45 μm polyvinylidene fluoride (PVDF) membranes (Merck Millipore, USA). Next the membranes were probed with primary rabbit polyclonal anti-E-cadherin, anti-N-cadherin antibodies (1:1000, both from Proteintech Group, USA) or rabbit anti-β-actin (1:1000, Sigma-Aldrich, Germany) antibody. Finally, analyzed proteins were detected with secondary anti-rabbit antibody conjugated with alkaline phosphatase (ECF Western Blotting Reagent Pack, GE Healthcare, Great Britain) and the signal was developed according to manufacturer’s instruction. Blots were visualized in Image Station 4000MM (Carestream Healthcare Inc., USA) and analyzed with ImageJ Software, as follows. The total E-cadherin cellular content comprising mature and unprocessed E-cadherin (with a molecular weight of approximately 100 and 130 kDa, respectively) was calculated. Then, E-cadherin and N-cadherin contents were normalized to β-actin. Finally, E-cadherin to N-cadherin ratios in individual samples were calculated and presented as median values.

### Systemic toxicity of the anti-cancer treatment

The toxicity of the proposed anti-cancer treatment strategy and its influence on the overall health condition was estimated on the basis of body weight changes as well as morphological blood analyzes. The body weight of experimental animals was measured three times each week throughout the course of all studies. Body weight changes were calculated using the following formula:

$$ \%\ BW=\frac{W_N}{W_0}\times 100-100 $$

where: W_N_ is the mean body weight of the animals on each successive day of the experiment; W_0_ is the mean body weight of the animals calculated for day 0 of the experiment.

### Statistical analysis

Data normality was estimated using the Shapiro-Wilk test with a predetermined *p* < 0.05 value. The Tukey-Kramer multiple comparisons test for parametric data or the Kruskal Walis Test for non-parametric data were applied; *p* values lower than 0.05 were considered significant. All calculations were performed using STATISTICA 10 (StatSoft Inc., USA) or GraphPad Prism 6 (GraphPad Software, Inc., USA) software.

Unless stated otherwise all data presented on graphs correspond to median values ± min/max.

## Results

### Anti-cancer activity of 1-MNA and 1,4-DMP in a single drug therapy

**Fig. 1:**
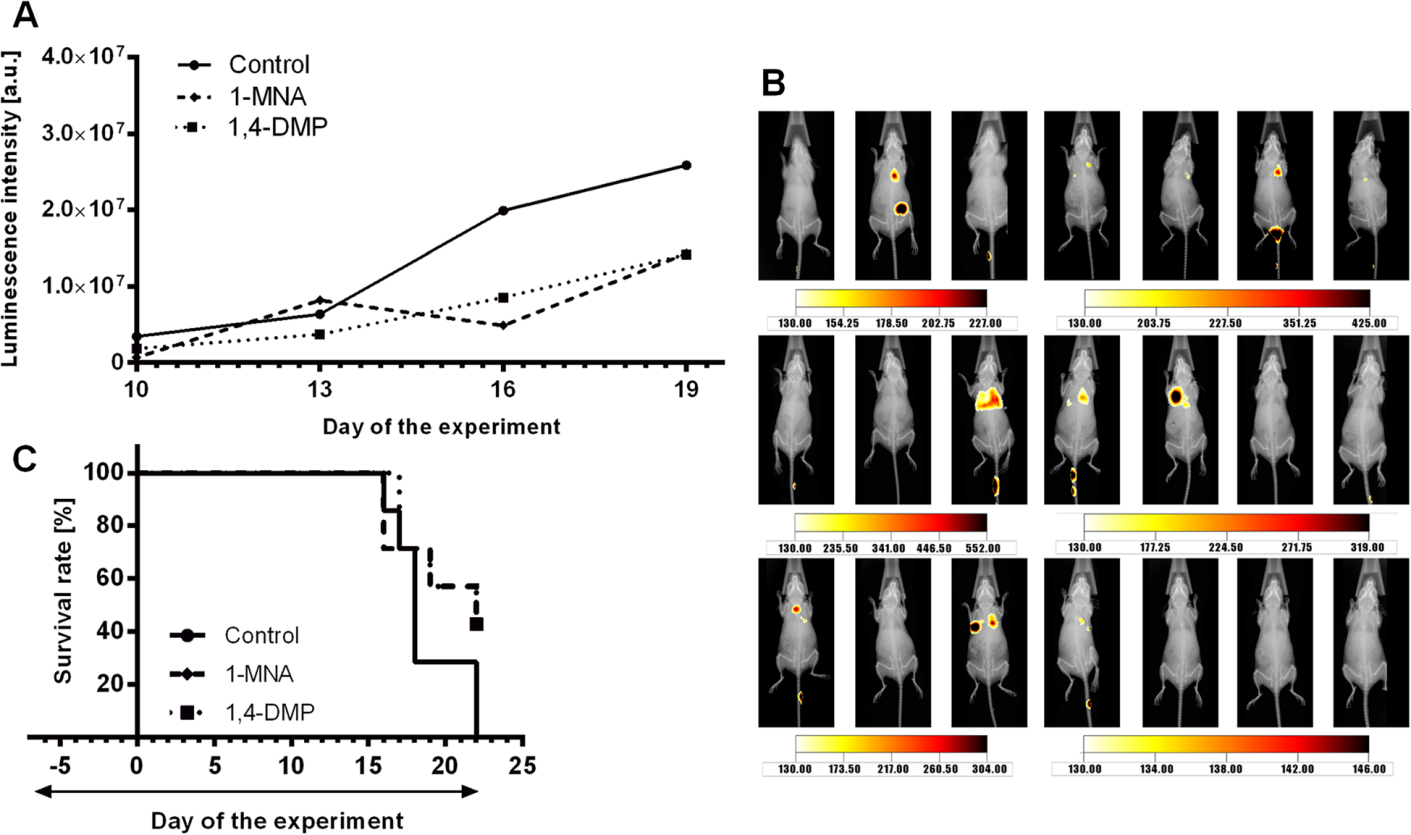
Anti-metastatic efficacy of 1-MNA and 1,4-DMP in the model of experimental metastasis of 4T1-luc2-tdTomato cells. **a** Luminescence intensity of 4T1-luc2-tdTomato cells visualized during intravital imaging of mice treated with 1-MNA and 1,4-DMP (100 mg/kg/day in a drinking water) from the 7th day prior cancer cells intravenous transplantation. Data are presented as mean values ± SD. **b** Images of luminescence intensity taken on the 10th day of the experiment for untreated animals (*upper row*), mice treated with 1-MNA (*middle row*), mice treated with 1,4-DMP (*bottom row*). **c** Kaplan-Meier survival curves obtained for the animals treated with 1-MNA and 1,4-DMP (days of administration are indicated on the graph with an arrow)

To assess the potential ability of 1-MNA and 1,4-DMP to inhibit metastases formation 4T1-luc2-tdTomato cells were intravenously inoculated into female BALB/c Nude mice that were pretreated with 1-MNA or 1,4-DMP for 7 days before transplantation. In vivo visualization of the metastatic lesions in lungs revealed that administration of either 1-MNA or 1,4-DMP resulted in delayed onset of metastatic lesion formation in mice (on the 10th day of the experiment metastatic lesions were observed in 3 out of 7 treated animals, whereas 5 out of 7 animals were diagnosed with lung metastases in the control group; Fig. 1b) and retarded metastases growth as estimated by means of intravital imaging of the lesions localized in lungs (Fig. 1a). When compared to the control group of mice the survival rate among the 1-MNA or 1,4-DMP-treated animals was increased by approximately 30 % on the last 22nd day of the experiment (in both of the groups 3 out of 7 animals survived until 22nd day of the experiment^,^ while there were no surviving animals in the control group, Fig. 1c). Prolonged treatment with 1-MNA and 1,4-DMP did not induce any side effects as during the whole experiment no symptoms of treatment related toxicity was observed (Additional file 1: Figure S1).

**Fig. 2:**
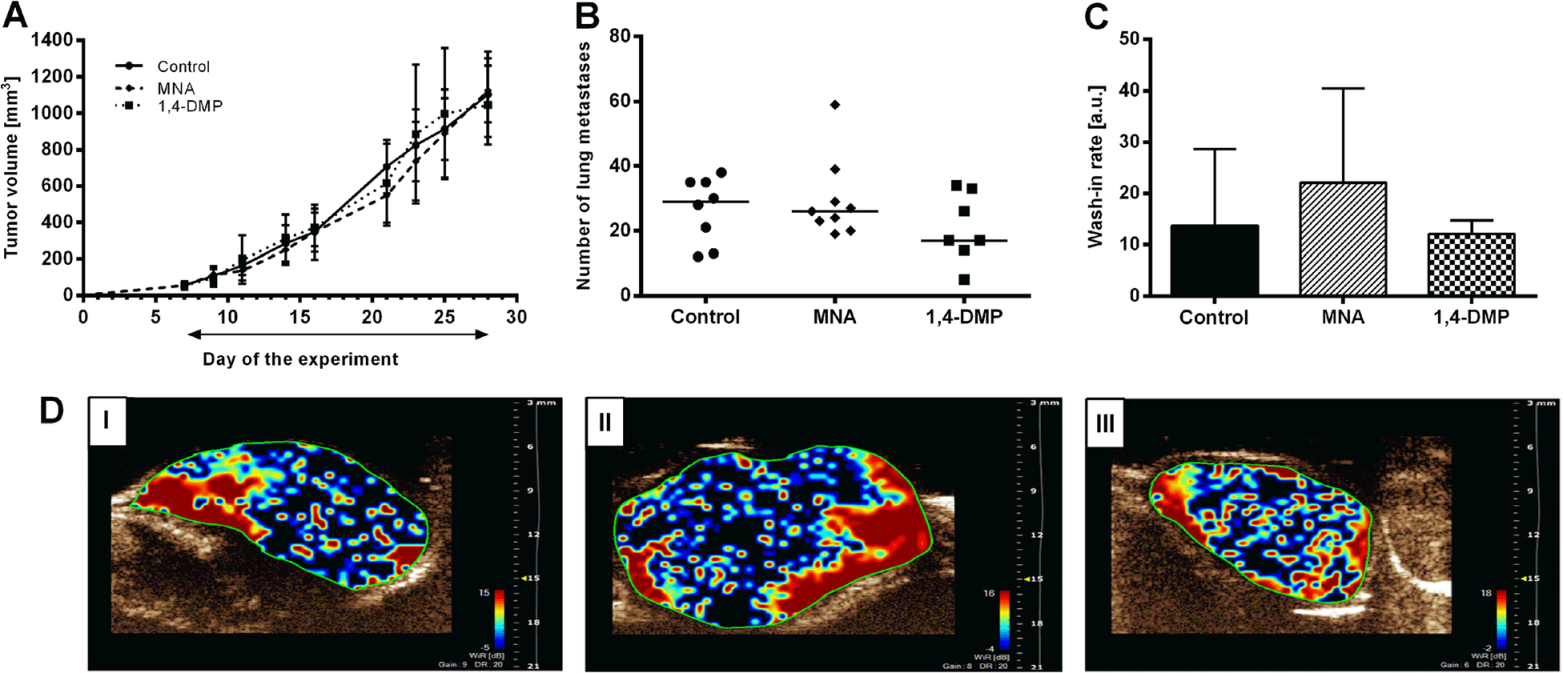
The influence of 1-MNA and 1,4-DMP on the growth, angiogenesis and metastasis of 4T1 tumors. **a** Tumor growth kinetics in mice treated with 1-MNA or 1,4-DMP given at the dose of 100 mg/kg/day in drinking water from the 7th day of the experiment (administration period is marked on the graph with an arrow). Data are presented as mean values ± SD. **b** Median number of lung metastases in BALB/c mice bearing 4T1 tumors. Data are presented as raw values with medians. **c** Wash-in-rate values estimated by the ultrasound imaging of 4T1 tumor blood perfusion performed on the 25th day of the experiment. Data are presented as median values ± min/max. **d** Representative images of tumor perfusion taken for: (I) control group, (II) mice treated with 1-MNA and (III) mice treated with 1,4-DMP

Since both 1-MNA and 1,4-DMP, while being well tolerated and non-toxic, featured anti-metastatic properties, their anti-cancer activity was further evaluated in the model of spontaneously metastasizing 4T1 murine mammary gland cancer. As presented in Fig. 2a, when administrated from the 7th day of the experiment, none of the tested compounds influenced the growth kinetics of 4T1 primary tumors transplanted into syngeneic BALB/c mice. Despite the lack of the anti-tumor activity, 1,4-DMP inhibited the formation of lung metastases by about 40 % in comparison to the control (17 vs. 29 median number of lung metastases, respectively, Fig. 2b). Additionally, the influence of both 1-MNA and 1,4-DMP on tumor angiogenesis was estimated. The results of the intravital ultrasound imaging of blood flow in tumor tissue reflected in wash-in rate parameter values indicated that 1,4-DMP did not influence tumor blood vessel formation (Fig. 2c, 2d(III)). 1-MNA, in turn, did not reveal any marked anti-metastatic activity (Fig. 2b); however, surprisingly it increased the value of the wash-in rate parameter by 40 % when compared to the control group of animals (the median value of wash-in rate expressed in arbitrary units [a.u.] was estimated to be 22.1 *vs*. 13.6, respectively, Fig. 2c, 2d (II)).

**Fig. 3:**
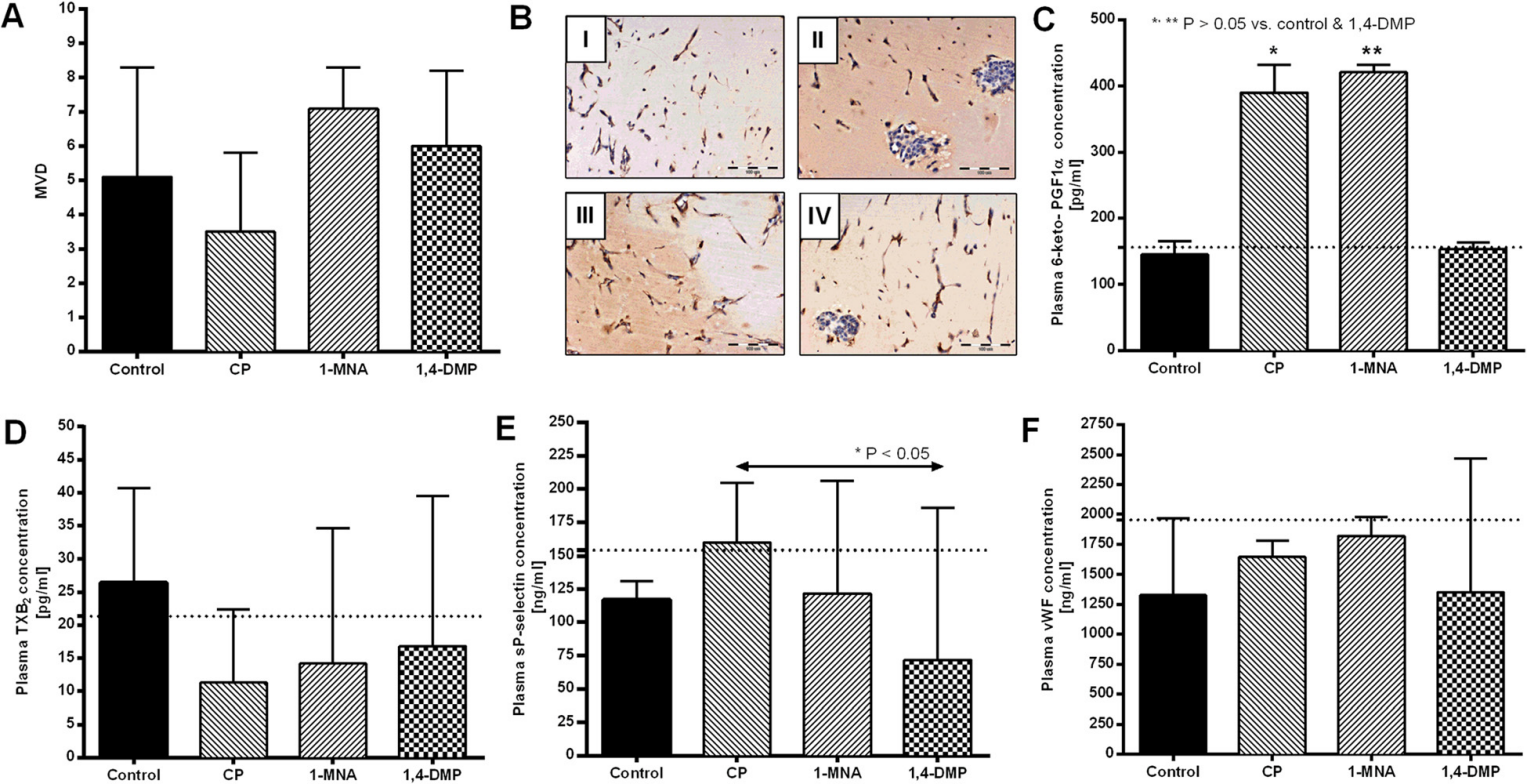
The influence of 1-MNA and 1,4-DMP on tumor angiogenesis estimated in the matrigel plug assay. **a** Mean microvessel density (MVD) quantified in matrigel plug sections collected on the 7th day after their implantation and stained with anti-CD31 antibody. Data are presented as median values ± min/max. **b** Images of sections stained with anti-CD31 antibody for untreated animals (I), mice treated with cyclophosphamide (CP) at the dose of 25 mg/kg given intraperitoneally on days 0, 3 and 5 (II), mice treated with 1-MNA (III) and mice treated with 1,4-DMP (IV). Both 1-MNA and 1,4-DMP were given at the dose of 100 mg/kg/day in drinking water, starting from the day of matrigel plug implantation. Plasma levels of: **c** 6-keto-PGF1α, **d** TXB_2_, **e** soluble P-selectin, **f** vWF in mice treated with cyclophosphamide, 1-MNA or 1,4-DMP; all data are presented as median values ± min/max, dotted lines on the graphs correspond to the value of the median concentration determined for respective agents in healthy BALB/c mice

To confirm the pro-angiogenic activity of 1-MNA observed in the model of the spontaneous metastasis of 4T1 tumors, the effect of 1-MNA and 1,4-DMP on tumor angiogenesis was additionally analyzed in the model of 4T1 cancer-cell induced angiogenesis utilizing matrigel plugs. Cyclophosphamide given in a metronomic regime served in this study as a control anti-angiogenic treatment and proved its efficacy, resulting in about a 30 % lower mean microvessel density (Fig. 3a and 3b(II)) when compared to the control group (Fig. 3a and 3b(I)) (MVD of 3.5 vs. MVD of 5.1, respectively). Interestingly, 1-MNA tended to enhance blood vessel formation, as shown by the median microvessel density in sections obtained on the 7th day of the experiment (MVD of 7.1, Fig. 3a and 3b(III)), while 1,4-DMP did not influence the angiogenesis (MVD of 6, Fig. 3a and 3b(IV)). The increased pro-angiogenic activity of 1-MNA was accompanied by a significant increase in 6-keto-PGF1α plasma concentration (median value of 420.5 pg/ml vs. 145.2 pg/ml in a control group of animals). Similarly, elevated 6-keto-PGF1α concentration was observed in the plasma samples obtained from mice treated with cyclophosphamide (390.3 pg/ml), while this was not noted in mice treated with 1,4-DMP (Fig. 3c). In contrast, in all treated groups reduced TXB_2_ plasma levels were observed (Fig. 3d), while plasma concentrations of vWF and soluble P-selectin were not significantly modified by 1-MNA or 1,4-DMP as compared to control (Fig. 3e–f).

### Anti-cancer activity of 1-MNA and 1,4-DMP in a combined treatment with cyclophosphamide

**Fig. 4:**
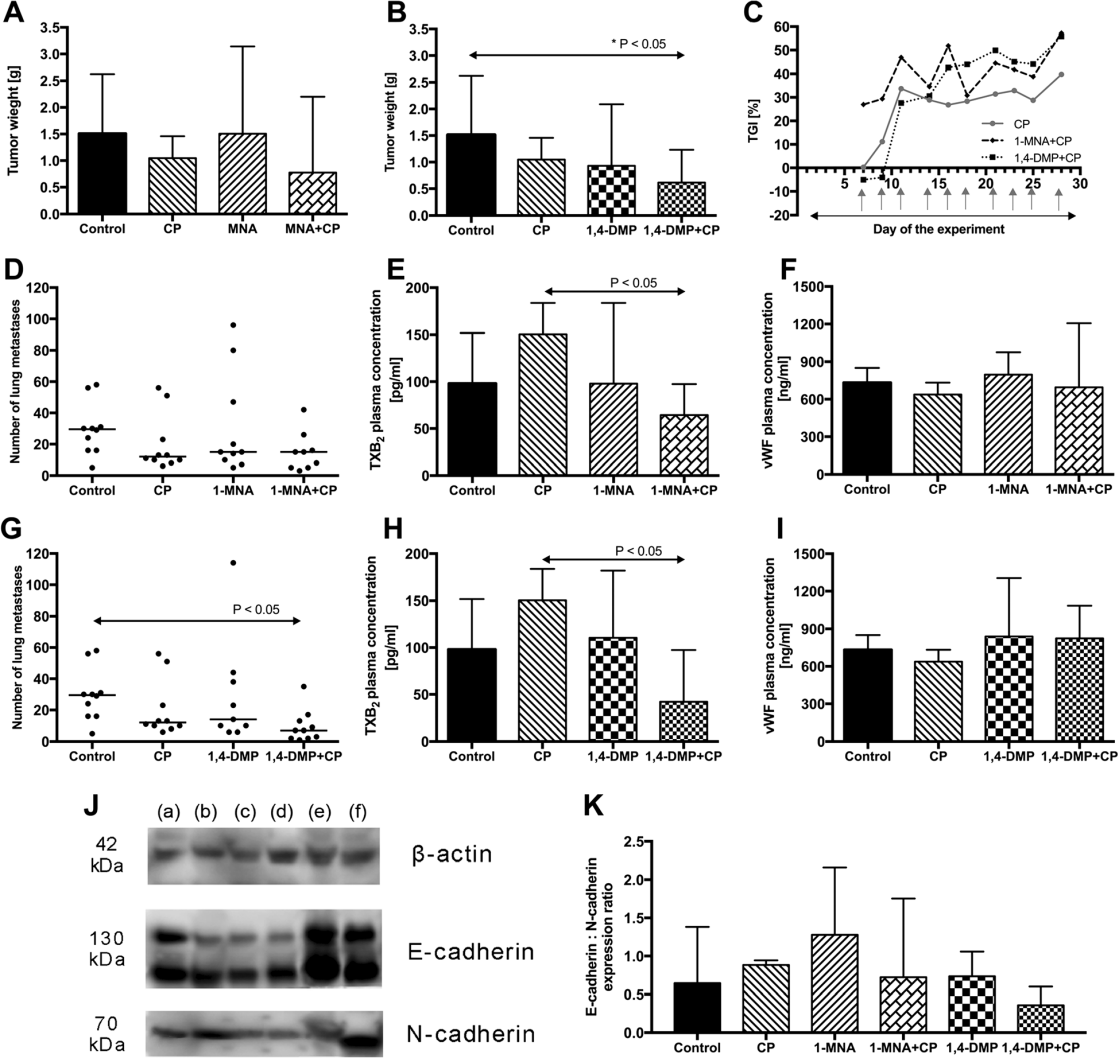
Anti-cancer efficacy of a combined treatment regime composed of 1-MNA or 1,4-DMP and cyclophosphamide in the 4T1 cancer model. For ease of interpretation, graphs are presented for the two therapeutic regimes separately. Tumor weight estimated on the 29^th^ day of the experiment for mice treated with **a** 1-MNA and cyclophosphamide (CP) or **b** 1,4-DMP and cyclophosphamide (CP) both presented with relevant controls; data are presented as median values  ± min/max. **c** Tumor growth inhibition (TGI) values observed for combined therapeutic regimes presented in reference to TGI of cyclophosphamide alone. In Fig. 4C, days of the drug administrations are indicated with arrows (1-MNA and 1,4-DMP - solid arrow, cyclophosphamide – gray arrows). The number of lung metastases in mice receiving **d** 1-MNA and cyclophosphamide (CP) or **g** 1,4-DMP and cyclophosphamide (CP) presented with corresponding controls; data are presented as raw values with medians. TXB_2_ plasma levels in mice receiving **e** 1-MNA and cyclophosphamide (CP) or **h** 1,4-DMP and cyclophosphamide (CP); data are presented as median values ± min/max. vWF plasma levels in mice receiving **f** 1-MNA and cyclophosphamide (CP) or **i** 1,4-DMP and cyclophosphamide (CP); data are presented as median values ± min/max. **j** The images of bands obtained in Western blot analysis of tissue samples isolated from the animals assigned to treatment with: (*a*) control, (*b*) cyclophosphamide (CP), (*c*) 1-MNA, (*d*) 1-MNA and cyclophosphamide (CP), (*e*) 1,4-DMP, (*f*) 1,4-DMP and cyclophosphamide (CP). **k** E-cadherin : N-cadherin expression ratios in the samples of tumor tissue collected on the last day of the experiment. The total cellular content of E-cadherin (comprising protein characterized by the molecular weight of 100 and 130 kDa) and N-cadherin was first normalized to the content of β-actin and then used to determine E-cadherin to N-cadherin expression ratios that are presented on the graph as median values with min/max

Due to the observed effects of 1-MNA and 1,4 DMP on tumor metastasis and angiogenesis, we next decided to investigate the influence of both of the compounds on the anti-cancer efficacy of cyclophosphamide applied in a metronomic dosage regime. The analysis of the primary 4T1 tumor growth kinetics as well as the weight of the tumor mass on the last (29th) day of the experiment indicated that both 1-MNA and 1,4-DMP enhanced the observed anti-tumor activity of cyclophosphamide (median tumor weight in the groups given cyclophosphamide with 1-MNA or 1,4-DMP was estimated as 0.78 g and 0.61 g, respectively, while the tumor weight in group of mice treated with cyclophosphamide alone was 1.05 g), which in the case of the latter resulted in a statistically significant tumor growth inhibition when compared to the control group of animals (tumor weight of 1.51 g, Fig. 4a–c). In contrast, 1-MNA in a combination with cyclophosphamide retained the anti-metastatic activity of the cytostatic drug given alone resulting in about a 60 % inhibition of lung metastases formation (12 vs. 29 median number of lung metastases in cyclophosphamide and control group, respectively) (Fig. 4d, 4g). However, when mice were treated with cyclophosphamide given with 1,4-DMP, a statistically significant inhibition of lung metastases formation was achieved. The median number of lung metastases (7 lung metastases) was about 80 % lower when compared to the control (29 lung metastases) and about 50 % lower when compared to the group treated with cyclophosphamide given in a single drug therapy (12 lung metastases, Fig. 4g). We also observed that TXB_2_ plasma level was decreased in those groups with the least number of identified metastases; however, vWF concentration remained at a similar level in all treated animals (Fig. 4e–f and 4h–i).

Interestingly, in tumor tissue isolated from mice treated with 1-MNA alone or in combination with cyclophosphamide, a higher E-cadherin expression that was accompanied with the lower N-cadherin levels was observed. The estimated E-cadherin to N-cadherin ratio in the tissue was more than twice as high as in the control untreated animals. On the other hand, such phenomena did not occur in mice receiving 1,4-DMP either in a single or combined treatment regime (Fig. 4j–k).

### Toxicity of the anti-cancer treatment

**Fig. 5:**
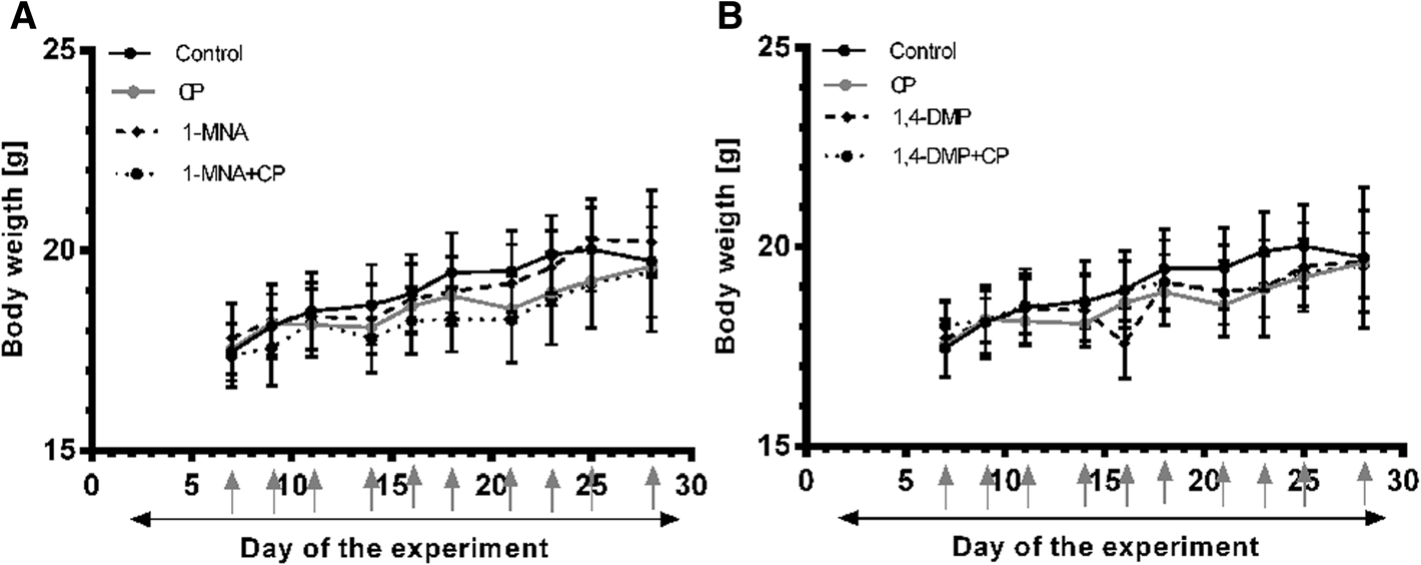
Body weight of BALB/c mice bearing 4T1 tumors, treated with **a** 1-MNA and cyclophosphamide (CP) or **b** 1,4-DMP and cyclophosphamide (CP), both presented with relevant controls. In Fig. 5a-b, days of the drug administrations are indicated with arrows (1-MNA or 1,4-DMP- *solid arrow*, cyclophosphamide – *gray arrows*). Data are presented as mean values ± SD

Neither of the treatments employed influenced the overall wellbeing of the animals as evidenced by the weight increase observed throughout the whole experiment (Fig. 5a–b). Therapeutic regimes applied in the study did not induce any significant changes in blood morphological parameters that might be attributed to toxic side effects. No cases of treatment-related deaths were recorded.

## Discussion

Anti-thrombotic, anti-inflammatory, and vasoprotective properties of 1-MNA ascribed to its contribution to PGI_2_ release have been proposed to have therapeutic significance, for example, in atherosclerosis [44]. On the other hand, when studied for its anti-metastatic properties, PGI_2_, which is the most potent endogenous inhibitor of platelet aggregation, has proven to be an efficient metastasis inhibitor [23, 45]. Stable analogs of PGI_2_ also displayed anti-cancer activity [38, 40–42]. However, the short lifetime of PGI_2_ and the side effects of its analogs when given systemically may limit their therapeutic application in oncology. Therefore, a PGI_2_-releasing agent, such as 1-MNA or its structural analog 1,4-DMP, might become an interesting alternative to the use of PGI_2_ in the treatment of metastatic cancer. Here, we have demonstrated that in the murine model of metastatic breast cancer 1-MNA and 1,4-DMP both display anti-metastatic activity, particularly during the vascular phase of metastasis and if combined with cyclophosphamide may ensure an efficient strategy for the prevention of mammary gland tumor’s metastases formation.

In two separate in vivo studies, both 1-MNA and 1,4-DMP inhibited to some degree the spontaneous formation of 4T1 tumor lung metastases while neither of them influenced the growth of 4T1 primary tumors. Moreover, the observed anti-metastatic effect was strongly related to the time of therapy initiation, being more pronounced while 1-MNA and 1,4-DMP were administrated from the time of 4T1 cells orthotopic transplantation. This, in turn, suggests that the activity of both compounds results in the prevention of early 4T1 tumor’s metastases formation rather than in any direct influence on established lesions. Such anti-metastatic activity of 1-MNA and 1,4-DMP was further confirmed in the model of experimental metastasis of 4T1-luc2-tdTomato cells that stably express red fluorescent protein and the gene for firefly luciferase. In the study, mice pretreated with either 1-MNA or 1,4-DMP revealed less pronounced metastatic disease characterized by a delayed onset indicating impaired 4T1 cells homing in the lungs. Such results remain in agreement with the previously disclosed anti-metastatic activity of 1-MNA and 1,4-DMP [26] and other published studies suggesting that drugs affecting platelet aggregation or endothelial inflammation might beneficially influence the vascular stage of metastatic dissemination, including adhesion of migrating cancer cells to the vessel wall and subsequent extravasation to the lungs [46, 47].

Possible implementation of 1-MNA and 1,4-DMP in the therapy of metastatic breast cancer was further confirmed when both compounds were administrated with cyclophosphamide to 4T1 tumor-bearing mice. Interestingly, in the applied tumor model not only did 1-MNA and 1,4-DMP inhibit lung metastases formation but also they beneficially influenced the anti-tumor activity of the cytostatic drug. Pronounced tumor growth inhibition in mice receiving the combined treatments was associated with diminished TXB_2_ plasma levels suggesting potential involvement of platelet-related mechanisms in the anti-cancer activity of both investigated molecules. Moreover, when given with cyclophosphamide, 1,4-DMP further improved the anti-metastatic efficacy of the chemotherapeutic.

Since metastases formation strongly depends on angiogenesis yield [48], we additionally decided to estimate the influence of 1-MNA and 1,4-DMP on vessel formation in either mammary gland tumor mass or matrigel plugs. The results of the studies revealed surprisingly distinct effects of 1-MNA and 1,4-DMP on vasculature development. While 1-MNA stimulated the formation of blood vessels in both angiogenesis models employed, 1,4-DMP did not affect vasculature formation when compared to the control group of animals. Such an observation is not surprising in terms of the proven PGI_2_-dependent activity of 1-MNA; however, it might indicate that either the PGI_2_ releasing capacity of 1,4-DMP is lower as compared with 1-MNA, or there is another anti-metastatic mechanism of action that could be featured by this compound. Such an assumption is further supported by the observation that, while both agents seemed to inhibit platelet activity as manifested by the reduced plasma level of TXB_2_, only 1-MNA induced a profound increase in PGI_2_ concentration, as reflected in elevated 6-keto-PGF1α levels. It is known that PGI_2_ displays pro-angiogenic activity that has been previously shown both in vitro and in vivo, in tumor as well as in ischemia models [49, 50–52]. In fact, Osawa et al. has shown that in tumor endothelial cells (TECs) the expression of the receptor for PGI_2_ is upregulated, but also that TECs secrete higher levels of PGI_2,_ which in turn stimulates angiogenesis in an autocrine manner [52]. Accordingly, we suspect that observed in our study increased capillary formation, induced in response to the treatment with 1-MNA, resulted from PGI_2_-releasing activity of the compound. In case of our studies, however, such enhanced tumor angiogenesis did not lead to enhanced tumor growth or metastatic dissemination, but contrarily to lesser number of diagnosed metastases and increased efficacy of the applied chemotherapeutic. Possibly, such effect emerged from the phenomena of tumor vessels normalization that could occur in response to 1-MNA. Such effect is not unparalleled as the relation between vascular permeability and the drug transport efficiency into tumors has been previously described [53]. Also, Li et al. shows that the prevention of VEGF-A induced tumor vasculature leakage increase chemotherapy efficacy and what is even more important the number of distant metastases [54].

It has been previously described that in order to form distant metastasis immobile tumor cells undergo epithelial to mesenchymal transition (EMT), a process in which non-invasive cells loose their epithelial character and develop mesenchymal features to eventually acquire more invasive potential. It has been shown that on the molecular level, EMT is driven by phosphatidylinositol-3-kinase/Akt (PI3K/Akt) [55] and extracellular signal regulated kinase 1/2 (ERK1/2) [56] signaling pathways that when activated contributed to an enhanced metastasis [57]. Activation of PI3K/Akt and ERK1/2 in cancer cells eventually leads to the down-regulated E-cadherin and up-regulated N-cadherin expression, among others. Accordingly, decreased E-cadherin cellular content was shown to promote cancer cells motility and invasiveness in vitro and consequently to correlate with worsened survival prognosis in patients [58, 59]. In our study, however, enhanced E-cadherin to N-cadherin expression ratio was observed in mammary gland tumor tissue isolated only from mice treated with 1-MNA or 1-MNA given with cyclophosphamide. It seems probable that 1-MNA exerted its anti-metastatic activity on the primary mammary gland tumor level leading to diminished metastatic potential of 4T1 cells, while the activity of 1,4-DMP is not directly related to the tumor tissue. Such observation further supports the idea that 1-MNA and 1,4-DMP affect malignant breast cancer progression in different manners.

Besides the undeniable beneficial anti-metastatic activity of the combined treatment comprising either 1-MNA or 1,4-DMP and cyclophosphamide, its sustained low toxicity comparable to that revealed by the cytostatic drug given alone is probably the most crucial factor from the clinical point of view. The most undesirable side effect of a treatment implementing anti-platelet drugs is bleeding [60–62]. However, in our study we have not observed any incidence of treatment-related deaths. Moreover, the body weight of the treated animals was constantly increasing throughout the study. This might be a consequence of the PGI_2_ releasing activity of the employed agents. PGI_2_ has been shown to be a more potent inhibitor of platelet-platelet interactions than an inhibitor of platelet adhesion to damaged endothelium [63]; therefore, it seems to constitute a safe alternative for other non-selective platelet inhibitors. Moreover, the recently described beneficial hepatoprotective activity of 1-MNA and NMMT [64] might suggest its potential preventive activity also against liver damages induced by chemotherapy.

On the other hand, recently published data showing an elevated expression of NMMT in a number of human tumor types and its possible involvement in proliferation, tumorogenic capacity and invasiveness of cancer cells [2, 3, 52, 65] may cause major concerns when considering implementation of 1-MNA and its analogs into the anti-cancer therapeutic regimes. Not only NMMT but also 1-MNA itself was shown to promote cellular growth what in a study of Parsons et al. resulted in the prolonged survival and enhanced proliferation of neurobastoma cells in vitro [66]. However, in our preliminary studies we have established that neither 1-MNA nor 1,4-DMP promote the proliferation of breast, prostate or colon cancer cells in vitro, even at the concentrations up to 1000 μg/ml. On the contrary, we have observed that while 1-MNA at such concentration did not reveal any influence on the growth of tested cells, 1,4-DMP actually inhibited it in 20 % to almost 50 %, depending on the cell line tested (Additional file 1: Table S1). Additionally, as evidenced by the results presented above neither of studied pyridine compounds enhanced the growth of 4T1 primary tumors in vivo. Such discrepancy observed between the experimental results regarding the activity of NMMT in tumor cells is not uncommon. For example, while a number of reports indicate the correlation between the NMMT expression and the degree of malignancy [65] other suggest that tumors express high levels of NMMT that promotes early phase of tumorogenicity, but has to be down-regulated to enable metastasis. In fact, these reports attribute NNMT’s high expression to the prolonged patients’ survival [67–69]. The exact mechanisms involved in the effects of NNMT in cancer cells are not entirely clear, however, it is suspected that they depend on the regulation of the processes based on the availability of NA and methyl groups [70]. Accordingly, down-regulation of NMMT was shown to induce apoptosis in human breast cancer cells [3]. It was reported that 1-MNA might act as NMMT inhibitor [71] what at least in theory could increase the bioavailability of intracellular NA and thus endure the NMMT effect leading to the induced apoptosis and chemosensitization of cancer cells [72, 73]. However, in the light of the results presented here, it seems that a possible direct effect of 1-MNA on 4T1 murine mammary gland cells, if any, is neglectable as it did not result in an enhanced tumorogenesis or chemoresistance development. On the contrary, implementation of 1-MNA and its analogue 1,4-DMP to the treatment of metastatic mammary gland cancer has proven beneficial. Therefore, concerns regarding the use of 1-MNA in the anticancer therapies that may arise on the basis of recently published NMMT activity in cancer cells seem to be unjustified as pro-metastatic activity of NNMT seems to be related to the intracellular methylation status in cancer cells, while exogenous 1-MNA exerts its anti-inflammatory and anti-thrombotic properties in circulation and thus indirectly affect cancer cells metastasis.

## Conclusions

The results of the studies presented above indicate that 1-MNA and its analog, 1,4-DMP, might be beneficially implemented into the treatment of metastatic breast cancer to ensure a more comprehensive strategy of metastasis control than that based on chemotherapy alone. However, additional studies should be performed to explain their mechanism of action in more detail and to establish whether the observed anti-metastatic activity of both compounds is restricted only to breast cancer or whether it is applicable to a broader range of malignant tumors.

## Abbreviations

%BW, % of body weight; 1,4-DMP, dimethylpyridinium chloride; 1-MNA, 1-methylnicotinamide chloride; 6-keto-PGF1α, 6-keto-prostaglandin F1α; AU, arbitrary units; CLO, clopidogrel; COX-2, cyclooxygenase 2; CP, cyclophosphamide; EDTA, ethylenediaminetetraacetic acid; EMT, epithelial to mesenchymal transition; ERK1/2, extracellular signal regulated kinase 1/2; HBSS, Hanks Balanced Salt Solution; IL-4, interleukin 4; NA, nicotinamide; NADH, nicotinamide adenine dinucleotide; NADPH, nicotinamide adenine dinucleotide phosphate; NNMT, nicotinamide N-methyltransferase; PI3K/Akt, phosphatidylinositol-3-kinase/Akt; PS, pyridinium salts; PVDF, polyvinylidene fluoride; ROS, reactive oxygen species; rpm, round per minute; SDS, sodium dodecylosulphate; TCIPA, tumor cell induced platelet; TECs, tumor endothelial cells; TGI, tumor growth inhibition; TNF-α, tumor necrosis factor α; TV, tumor volume; TXB_2_, thromboxane B_2_; VCAM-1, vascular cell adhesion molecule 1; vWF, von Willebrand Factor

## Additional file

Additional file 1: Figure S1. Body weight of BALB/c Nude mice intravenously inoculated with 4T1-luc2-tdTomato cells treated with 1-MNA or 1.4-DMP in comparison to the control untreated group of animals. **Table S1. **The influence of 1-MNA and 1,4-DMP on the growth of selected cancer cell lines in vitro. (DOCX 1705 kb)
