# Supplementary material for: 1-methylnicotinamide and its structural analog 1,4-dimethylpyridine for the prevention of cancer metastasis
Source: J Exp Clin Cancer Res. 2016 Jul 13;35:110. doi: 10.1186/s13046-016-0389-9 (PMC4944260; doi:10.1186/s13046-016-0389-9)
Supplement: Additional file 1: Figure S1. — Body weight of BALB/c Nude mice intravenously inoculated with 4T1-luc2-tdTomato cells treated with 1-MNA or 1.4-DMP in comparison to the control untreated group of animals. Table S1. The influence of 1-MNA and 1,4-DMP on the growth of selected cancer cell lines in vitro. (DOCX 1705 kb) [file 13046_2016_389_MOESM1_ESM.docx]

**Supplementary data**

**1-methylnicotinamide and its structural analog 1,4-dimethylpyridine for the prevention of cancer metastasis**

Agnieszka Blazejczyk^1^, Marta Switalska^1^, Stefan Chlopicki^2,3^, Andrzej Marcinek^4^, Jerzy Gebicki^4^, Marcin Nowak^5^, Anna Nasulewicz-Goldeman^1^, Joanna Wietrzyk^1^

^1^ Hirszfeld Institute of Immunology and Experimental Therapy, Polish Academy of Sciences, Weigla 12, 53-114 Wroclaw, Poland; [agnieszka.blazejczyk@iitd.pan.wroc.pl](mailto:agnieszka.blazejczyk@iitd.pan.wroc.pl); [switalska@iitd.pan.wroc.pl](mailto:switalska@iitd.pan.wroc.pl);

[nasulewicz@iitd.pan.wroc.pl](mailto:nasulewicz@iitd.pan.wroc.pl); [wietrzyk@iitd.pan.wroc.pl](mailto:wietrzyk@iitd.pan.wroc.pl)

^2^ Chair of Pharmacology, Jagiellonian University, Medical College, Grzegórzecka 16, 31-531 Krakow, Poland

^3^ Jagiellonian Center for Experimental Therapeutics (JCET), Jagiellonian University Bobrzynskiego 14, 30-348 Krakow, Poland; [stefan.chlopicki@jcet.eu](mailto:stefan.chlopicki@jcet.eu)

^4^ Lodz University of Technology, Zeromskiego 116, 90-924 Lodz, Poland; [jerzy.gebicki@p.lodz.pl](mailto:jerzy.gebicki@p.lodz.pl); [andrzej.marcinek@p.lodz.pl](mailto:andrzej.marcinek@p.lodz.pl)

^5^ Wroclaw University of Environmental and Life Sciences, Norwida 31, 50-375 Wroclaw, Poland; [marcin.nowak@up.wroc.pl](mailto:marcin.nowak@up.wroc.pl)


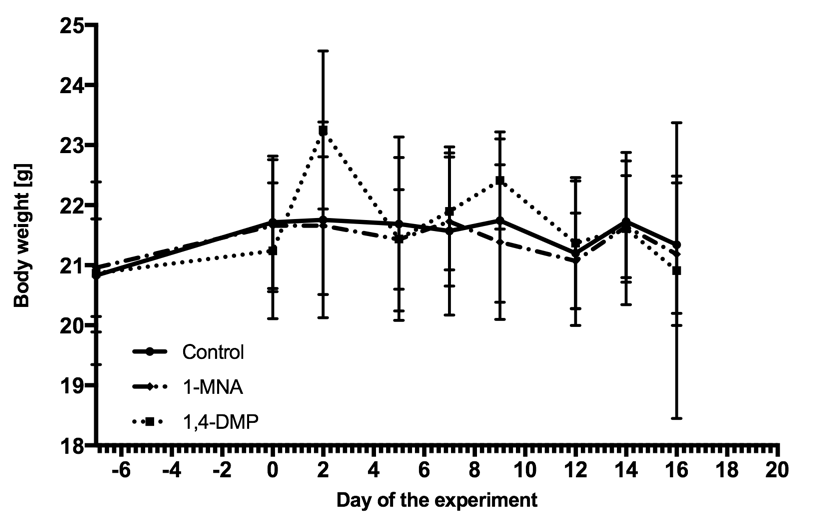


**Suppl. Figure 1. Body weight of BALB/c Nude mice intravenously inoculated with 4T1-luc2-tdTomato cells treated with 1-MNA or 1.4-DMP in comparison to the control untreated group of animals.**

**Suppl. Table 1: The influence of 1-MNA and 1,4-DMP on the growth of selected cancer cell lines *in vitro***

| **Tested cell line** | **Growth inhibition [%] *** | |
| --- | --- | --- |
|  | **1-MNA** | **1,4-DMP** |
| **4T1** | 5.25±1.78 | 35.58±17.58 |
| **4T1- luc2-tdTomato** | 0.78±0.51 | 44.19±23.53 |
| **MDA-MB-luc2-tdTomato** | 9.45±1.11 | 20.94±1.93 |
| **HT-29-luc2** | 2.58±2.82 | 48.46±5.92 |
| **PC-3M-luc2** | 0.72±1.25 | 22.19±1.53 |
| * The growth inhibition value was estimated after 72 hours exposure of the tested cells on 1-MNA and 1,4-DMP (both in the concentration of 1000 μg/ml). Data are presented as mean±SD.  MDA-MB-luc2-tdTomato – human breast cancer; HT-29-luc2 – human colon cancer; PC-3M-luc2 – human prostate: cancer cell lines stably expressing the firefly luciferase gene were obtained from Caliper Life Sciences Inc. (USA). | | |
